# *Lacticaseibacillus paracasei L21* and Its Postbiotics Ameliorate Ulcerative Colitis Through Gut Microbiota Modulation, Intestinal Barrier Restoration, and HIF1α/AhR-IL-22 Axis Activation: Combined In Vitro and In Vivo Evidence

**DOI:** 10.3390/nu17152537

**Published:** 2025-08-01

**Authors:** Jingru Chen, Linfang Zhang, Yuehua Jiao, Xuan Lu, Ning Zhang, Xinyi Li, Suo Zheng, Bailiang Li, Fei Liu, Peng Zuo

**Affiliations:** 1 Key Laboratory of Dairy Science, Ministry of Education, Food College, Northeast Agricultural University, Harbin 150030, China; chenjingru121@163.com (J.C.); 18537306353@163.com (X.L.); 15848981381@163.com (N.Z.); 15145007118@163.com (X.L.); zhengsuowork@outlook.com (S.Z.); 15846092362@163.com (B.L.); 2 Department of Grain Engineering, Heilongjiang Jiaotong Polytechnic, Harbin 161000, China; hjyzlf2018@163.com; 3 Center of Drug Safety Evaluation, Heilongjiang University of Chinese Medicine, Harbin 150040, China; jiaoyh@hljucm.edu.cn; 4 College of Arts and Sciences, Northeast Agricultural University, Harbin 150030, China

**Keywords:** lactic acid bacteria, ulcerative colitis, gut microbiota, inflammatory cytokines, intestinal barrier

## Abstract

**Background:** Ulcerative colitis (UC), characterized by chronic intestinal inflammation, epithelial barrier dysfunction, and immune imbalance demands novel ameliorative strategies beyond conventional approaches. **Methods:** In this study, the probiotic properties of *Lactobacillus paracasei* *L21* (*L. paracasei* *L21*) and its ability to ameliorate colitis were evaluated using an in vitro lipopolysaccharide (LPS)-induced intestinal crypt epithelial cell (IEC-6) model and an in vivo dextran sulfate sodium (DSS)-induced UC mouse model. **Results:** In vitro, *L. paracasei* *L21* decreased levels of pro-inflammatory cytokines (TNF-α, IL-1β, IL-8) while increasing anti-inflammatory IL-10 levels (*p* < 0.05) in LPS-induced IEC-6 cells, significantly enhancing the expression of tight junction proteins (ZO-1, occludin, claudin-1), thereby restoring the intestinal barrier. In vivo, both viable *L. paracasei* *L21* and its heat-inactivated postbiotic (H-L21) mitigated weight loss, colon shortening, and disease activity indices, concurrently reducing serum LPS and proinflammatory mediators. Interventions inhibited NF-κB signaling while activating HIF1α/AhR pathways, increasing IL-22 and mucin MUC2 to restore goblet cell populations. Gut microbiota analysis showed that both interventions increased the abundance of beneficial gut bacteria (*Lactobacillus*, *Dubococcus*, and *Akkermansia*) and improved faecal propanoic acid and butyric acid levels. H-L21 uniquely exerted an anti-inflammatory effect, marked by the regulation of *Dubosiella,* while *L. paracasei* *L21* marked by the *Akkermansia*. **Conclusions:** These results highlight the potential of *L. paracasei* *L21* as a candidate for the development of both probiotic and postbiotic formulations. It is expected to provide a theoretical basis for the management of UC and to drive the development of the next generation of UC therapies.

## 1. Introduction

Ulcerative colitis (UC), as a subtype of inflammatory bowel disease (IBD), consists of lesioned regions that mainly include the rectal, colonic mucosa, and submucosa [1]. UC’s pathogenesis is complex. It involves genetic factors, environmental factors (such as lifestyle and diet changes), and disruptions in the gut microbiome and its interactions with the host [2,3,4]. Overactive gut bacteria can cause dysfunction of the intestinal mucosa immune response, which contributes to the induction of UC [5]. However, the exact cause of UC remains unknown, which makes its control challenging. Currently, the global prevalence of UC is around 0.3% and China has the highest UC prevalence in Asia [6,7]. UC patients exhibit severe gastrointestinal symptoms. These symptoms require effective treatment with specific drugs (such as aminosalicylates, glucocorticoids, immunosuppressants, biologics, etc.), while in severe patients, surgical treatment may be required [6]. But both drugs and surgery may have serious side effects and high recurrence rates [8]. Therefore, the development of effective drug replacement therapies for UC is critical.

Recent studies show diet changes can affect the gut microbiome’s composition and function [9]. Probiotics are a common dietary approach targeting the gastrointestinal microbiome. Probiotics can boost immunity, correct gut microbiota abnormalities, reduce intestinal inflammation, and protect the intestinal barrier. These advantages make probiotics promising for preventing and managing UC [10]. Cytokines produced by resident cells in the gut induce the activation and stimulation of downstream immune dysregulation-associated pathways (Nuclear Factor kappa-B, NF-κB; Mitogen-Activated Protein Kinase, MAPK; Janus kinase-signal transducer and activator of transcription, JAK-STAT, etc.) by binding to cytokine receptors. Recently, a study indicated that the administration of *Lactobacillus acidophilus* and subsequent generation of ursodeoxycholic acid effectively alleviates inflammation in mice suffering from UC, which was achieved by activating various signaling pathways and adjusting the activity of regulatory T cells and M1 macrophages [11]. Moreover, some probiotic metabolites have immunomodulatory effects on the host. Short-chain fatty acids (SCFAs) have anti-inflammatory effects by influencing immune cell movement, and they also suppress inflammatory cytokine secretion and enhance interleukin-10 (IL-10) release [12,13]. SCFAs also strengthen the intestinal mucosa and inhibit lipopolysaccharide (LPS)-stimulated pro-inflammatory cytokine expression in immune cells [14]. However, probiotics do not completely manage gut microbiota. Studies have shown that when the immune system is weak, live probiotics can move from the intestinal lumen to the viscera and cause systemic infections [15]. Therefore, probiotics should be used cautiously in immunocompromised patients [16].

Postbiotics are a complex of inactivated microorganisms, bacterial components, and their metabolites. Postbiotics have a clear genetic background, benefit the health of the host, and are usually produced by heat-inactivating [17]. Compared to live probiotics, Postbiotics have the benefits of being safer and more stable. Postbiotics can resist gastrointestinal stress and be precisely dosed, which makes them safer for immunocompromised people and suitable for developing into pharmacodynamic biologics. Studies have reported that postbiotics can promote mucosal production and improve its function, thereby inducing the host’s immune system and anti-inflammatory response [18]. Postbiotics are beneficial for maintaining intestinal homeostasis, intestinal epithelial barrier function, and establishing intestinal homeostasis. Some studies have suggested using postbiotics in UC therapy [19]. Therefore, exploring probiotics and their postbiotics with intestinal barrier-protecting functions and potential anti-inflammatory capacity is important for the development of biologics to alleviate UC.

In this study, *L. paracasei* *L21*, with excellent fundamental probiotic capabilities and anti-inflammatory ability in vitro, was isolated from traditional fermented yak milks in Gansu province. The postbiotics were obtained by heat inactivation. LPS-induced mice intestinal crypt epithelial cell (IEC-6) in vitro and dextran sulfate sodium (DSS)-induced C57BL/6J mice colitis models in vivo were established. The aim of this study is to investigate the protective role of *L. paracasei* *L21* and its postbiotic metabolites in DSS-induced colitis, focusing on the mechanisms of restoration of physiological phenotypes, reduction of cytokine levels, and regulation of the gut microbiota. The application of probiotics and postbiotics in alleviating UC holds promise for exploring the disparities between their uses. This exploration can provide a theoretical foundation for the development of a novel postbiotic preparation to alleviate UC.

## 2. Materials and Methods

### 2.1. Chemicals and Reagents

Dulbecco’s Modified Eagle Medium (DMEM) was provided by Hyclone company (Logan, UT, USA). Lipopolysaccharide, bile salt, and Pepsin (1:10,000) were purchased from Beijing Boao Tuoda Technology Co., Ltd. (Beijing, China). Trypsin-Ethylenediaminetetraacetic Acid (EDTA) (1:250) was purchased from Gibco Life Sciences (New York, NY, USA). Bacterial Total DNA Extraction Kit was purchased from Tiangen Biochemistry Science and Technology Co. Ltd. (Beijing, China); Cell/Tissue Total RNA Extraction Kit and RNA Reverse Transcription Kit were purchased from Aidlab Biotechnologies Co., Ltd. (Beijing, China); interleukin-22 (IL-22), LPS, tumor necrosis factor (TNF-α), interleukin-8 (IL-8), interleukin-1β (IL-1β), and IL-10 kits were purchased from Chenglin Biotechnology Co. Ltd. (Beijing, China); myeloperoxidase (MPO) test kit was provided by Nanjing Jiancheng Technology Co. Ltd. (Nanjing, China); DSS was purchased from MP Biomedicals (Irvine, CA, USA), molecular weight of 36,000 to 50,000. Detailed information on the antibodies used for immunofluorescence, immunohistochemistry, and Western Blot is given in Table A4.

### 2.2. Isolation, Purification, and Identification of Strains

*Lacticaseibacillus rhamnosus* GG (LGG) was provided by the Dairy Industry Culture Collection of Key Laboratory of Dairy Science of the Northeast Agricultural University, Ministry of Education, China (KLDS-DICC). Other *Lactobacillus* strains were isolated and cultured from traditional Gansu yak milk samples stored in the laboratory. Characteristic colonies were selected for microscopic examination (Hitachi, Tokyo, Japan) and re-spread on plates for purification until pure strains were obtained. The selected strains were stored in glycerol tubes at −80 °C for use. A total of 21 lactic acid bacterial strains were isolated, numbered L21, L25, 29, B1, 1.0651, 1.0403, 30, L3, 1.0351, 1.0912, M1, M2, M23, M24, M25, 1.0318, 1.0134, 1.0320, 1.0316, 1.0317, and H10, and were stored in the KLDS-DICC. To obtain a 5 × 10^9^ CFU/mL *L. paracasei* *L21* suspension for cell treatment or gavage, activated probiotics were centrifugation at 4 °C, 8000× *g* for 10 min and resuspended in phosphate-buffered saline (PBS buffer). Heat-inactivated *L. paracasei* *L21* (H-L21) was prepared following the method of Chung et al. [20]. Briefly, the bacterial suspension was heat-treated in a water bath at 80 °C for 30 min to inactivate cells and obtain postbiotics.

The 16S rRNA and housekeeping gene *pheS* of *Lactobacillus* were selected for PCR amplification. Sanger sequencing was conducted by Shanghai Bioengineering Co., Ltd. (Shanghai, China) The 16S rRNA gene sequencing results were analyzed using BLAST+ 2.15.0 in GenBank, and the phylogenetic tree was constructed using the Neighbor-Joining method in Mega 7.0 software. All primer sequences are shown in Table A1.

### 2.3. Determination of Probiotic Properties of Lactobacillus Strains

To evaluate the probiotic potential of *Lactobacillus* strains, a series of functional tests was conducted, and minor modifications based on previous methods, including gastrointestinal tolerance [21], cell surface hydrophobicity [22], and auto-aggregation capacity [23].

Gastrointestinal tolerance test: the activated *Lactobacillus* strains were cultured in MRS broth at 37 °C for 18 h, followed by centrifugation (4 °C, 8000× *g*, 10 min) to harvest the cells. The cells were washed twice with sterile PBS and resuspended in artificial simulated gastric fluid (pH 2.5) at a concentration of 1 × 10^9^ CFU/mL. After incubation at 37 °C for 3 h, viable cell counts were determined using the plate colony counting method. The cells were then recovered by centrifugation, resuspended in artificial simulated intestinal fluid (pH 8.0), and incubated for an additional 3 h, after which viable counts were again determined. The survival rate was calculated as follows:

(1) $$\mathrm{Survival}\;\mathrm{rate}\;(\%)\;=\;\log A/\log A_{0}\;\times \;100,$$

where *A* is the CFU count after treatment with simulated gastric/intestinal fluid, and *A*_0_ is the initial CFU count.

Cell surface hydrophobicity assay: The bacterial suspension (1 × 10^9^ CFU/mL in PBS) was mixed with xylene (1:3 *v*/*v*), vortexed for 120 s, and allowed to phase-separate for 1 h. The aqueous phase was collected, and its optical density (OD) at 600 nm (*OH*_1_) was measured using a UV spectrophotometer. The hydrophobicity (%) was calculated as follows:

(2) $$\mathrm{Cell}\;\mathrm{surface}\;\mathrm{hydrophobicity}\;(\%)\;=\;[({OH}_{0}\;-\;{OH}_{1})/{OH}_{1}]\;\times \;100,$$

where *OH*_0_ is the OD600 of the initial bacterial suspension.

Auto-aggregation capacity measurement: The bacterial suspension (1 × 10^9^ CFU/mL in PBS) was incubated at 37 °C for 3 h and 24 h. The OD600 values of the suspension were measured before (*D*_0_) and after incubation (*Dₜ*). The auto-aggregation capacity (%) was calculated as follows:

(3) $$\mathrm{Automatic}\;\mathrm{aggregation}\;\mathrm{capacity}\;(\%)\;=\;(1\;-\;D_{T}/D_{0})\;\times \;100,$$

### 2.4. Cell Culture and Treatment

IEC-6 cells, obtained from Zhejiang Meisen Cell Technology Co., Ltd. (Hangzhou, China), were cultured in DMEM supplemented with 10% fetal bovine serum (FBS) and 1% penicillin/streptomycin. The cells were maintained at 37 °C in a humidified incubator with 5% CO_2_. To induce inflammation, the cells were stimulated with 10 μg/mL LPS for 24 h. After LPS treatment, the cells were treated with the *Lactobacillus* strains (resuspended in antibiotic-free DMEM) at a multiplicity of infection (MOI) of 10:1 and incubated for 24 h. IEC-6 cells (1.0 × 10^5^ CFU/mL) were inoculated into 96-well cell culture plates and the effects of different concentrations of LPS, tested strains, or MOI on the viability of IEC-6 cells were assessed by the CCK-8 assay. The cell viability (%) was calculated as follows:

(4) $$\mathrm{Cell}\;\mathrm{viability}\;(\%)\;=\;(\frac{{test\;group}_{OD450\mathrm{nm}}-{\;blank\;group}_{OD450\mathrm{nm}}}{{control\;group}_{OD450\mathrm{nm}}-\;{blank\;group}_{OD450\mathrm{nm}}})\;\times \;100,$$

TNF-α secretion from IEC-6 cells stimulated by LPS was detected following the instructions of the ELISA kit (Chenglin Biotechnology Co. Ltd., Beijing, China).

### 2.5. Animals Experimental Design

C57BL/6J male mice (6 weeks old, n = 50) were purchased from Liaoning Changsheng Biotechnology Co., Ltd. (License number: SCXK (Liao) 2020-0001, Shenyang, China). All animal experimental procedures were reviewed and approved by the Animal Ethics Committee of Northeast Agricultural University (No. NEAUEC20230403, 9 November 2023). Mice were group-housed in a strictly controlled environment with a temperature of 22 ± 2 °C and a humidity of 55% ± 5%, and provided free access to food and water under a 12-h light–dark cycle. Mice were fed a purified standard diet AIN-93 purchased from Trofi Feed Technology Co., Ltd. (Nantong, China), refer to Table A2 for detailed information. During the gavage period, cage positions were rotated weekly (5 pcs/cage) to control for potential environmental effects, and feeding schedules were standardized. After 4 days of acclimatization to the environment, with reference to previous research protocols, the experimental animals were randomly divided into 5 groups using the completely random grouping method, with 8–10 mice per group [24].

**Figure A1:**
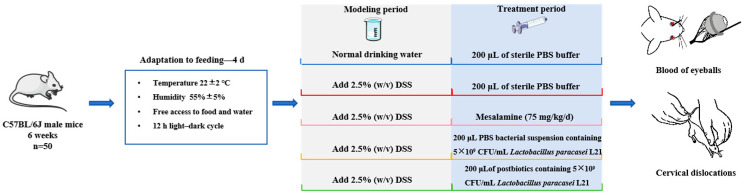
Animal experimental design.

The 14-day experiment was divided into a 7-day modeling period and a 7-day treatment period (Figure A1). Mice in the normal control (NC) group had free access to normal water, while the remaining groups were given water containing 2.5% (*w*/*v*) dextran sulfate sodium (DSS) to induce acute colitis. In the treatment period, mice in the NC group received 200 μL of sterile PBS buffer by intragastric administration daily. The DSS group received 200 μL of sterile PBS buffer. The G group was given mesalazine at a dose of 75 mg/kg/d. The L group was administered 200 μL of a 5 × 10^9^ CFU/mL *L. paracasei* *L21* suspension, and the H group received 200 μL of postbiotics (prepared from 5 × 10^9^ CFU/mL of *L. paracasei* *L21*). Animals treated with DSS showed signs of severe disease associated with colitis, such as blood in the stool and weight loss. Weight loss of more than 20% of total body weight with hunched posture and/or inactivity was defined as a humane endpoint. Mice or samples with unexpected deaths caused by DSS modeling, improper gavage handling, or sample processing failures were excluded from analysis. During the course of this experimental study, no animal deaths other than euthanasia were recorded.

Each mouse was treated independently and analyzed as a separate sample. The fecal consistency and degree of blood in the stool were weighed and recorded daily during the treatment period, which were then scored based on the severity of the condition. Table A3 displays the disease activity index (DAI) scale, which follows this formula: DAI = Fecal pattern score + stool occult blood score + weight loss score. At the end of the experimental period (day 15), mice were anesthetized by intraperitoneal injection of 75 mg/kg pentobarbital sodium. Blood samples were collected from the eyes, and the mice were euthanized by cervical dislocation. Subsequently, serum, colon tissue, spleen, and intestinal content samples were collected for the calculation of the mice splenic index and other analyses. The MPO activity in colonic tissues was evaluated using a biochemical kit. All samples were stored at −80 °C for use.

### 2.6. Immunofluorescence

Immunofluorescence was used to detect tight junction protein changes in IEC-6 cells. IEC-6 cells were inoculated into 6-well plates and cultured for 24 h. Next, the cells were fixed with 75% alcohol at room temperature (RT) for 20 min, permeabilized with membrane-breaking solution (10 min), and blocked with 10% goat serum (30 min). Cells were incubated with primary antibody overnight at 4 °C, then with secondary antibody for 50 min in the dark. After being washed three times with PBS (pH 7.4), DAPI was added, incubated for 10 min (dark, RT), and cells were mounted with anti-fluorescence quenching for fluorescence microscopy and imaging. The immunofluorescence was analyzed using Image-pro plus 6.0 (Media Cybernetics, Inc., Rockville, MD, USA) and results expressed as integrated optical density (IOD).

### 2.7. Immunohistochemistry

Four-micrometer colon paraffin sections were deparaffinized in xylene, hydrated in gradient ethanol, and antigen-retrieved by microwaving in citrate buffer (pH 6.0). Next, they were treated with 3% hydrogen peroxide for 25 min (dark, RT), blocked with 3% BSA for 30 min, incubated with primary antibody overnight, then with secondary antibody for 50 min. After DAB development, nuclei were counter-stained with hematoxylin. Sections were dehydrated, cleared in xylene for 5 min, and observed under a microscope. IHC scores for proteins are measured using the IHC profile in the ImageJ (v.1.54) program.

### 2.8. Quantitative Reverse Transcription Polymerase Chain Reaction (qRT-PCR)

Total RNA was extracted using the Total RNA Extraction Kit (for IEC-6 cells) and the Animal Tissue Total RNA Extraction Kit (for colon tissue) following the instructions provided by Beijing Adderall Bioscience and Technology Co., Ltd. (Beijing, China). Subsequent steps were performed according to the instructions of the reverse transcription kit. qRT-PCR analysis was then performed by using the quantitative real-time PCR kit. Reference genes (GAPDH for tissues/β-actin for cells) were designed via Primer 5.0 software (Jilin Kumai Biotechnology, Jiaohe, China) and specific primer sequences are detailed in Table A4.

### 2.9. Inflammatory Cytokine

The levels of TNF-α, IL-1β, IL-8, IL-10, IL-22, or LPS in cell supernatants, colon tissue homogenates, and serum samples were determined using the ELISA kit (Chenglin Biotechnology Co Ltd., Beijing, China) according to the manufacturer’s instructions.

### 2.10. Morphological Analysis

The method was slightly modified according to the description by Dieleman et al. [25]. In short, the colon tissues were dehydrated with ethanol until transparent and then embedded in paraffin. The embedded specimens were cut into 4 μm thin slices, stained with hematoxylin–eosin (H&E) and alcian blue (AB-PAS), sealed in neutral gum, and observed microscopically, and images were collected.

### 2.11. Western Blot

Western blotting analysis followed the method previously outlined [26]. Colon tissue proteins were homogenized in ice-cold RIPA lysis buffer supplemented with protease inhibitor cocktail (100:1 *v*/*v*) and PMSF, followed by centrifugation at 13,000× *g* (4 °C, 5 min). Protein concentrations in supernatants were quantified using a BCA assay with bovine serum albumin standards. Samples were denatured in 5× SDS loading buffer (4:1 ratio) at 95 °C for 10 min. Equal protein (10 μg/lane) underwent electrophoretic separation on 10% SDS-PAGE gels (Mini-PROTEAN^®^ system, Bio-Rad, Hercules, CA, USA) at 80 V (30 min) for stacking and 120 V (60 min) for resolution. Proteins were transferred to 0.45 μm PVDF membranes. The membranes were reversibly stained with Ponceau S, then blocked with 5% non-fat milk in TBST (2 h, 25 °C). Primary antibodies (diluted in TBST with 1% BSA) were incubated overnight at 4 °C, followed by 1 h incubation with HRP-conjugated secondary antibodies (37 °C). After three TBST washes (5 min each), immunoreactive bands were visualized using ECL colour rendering and quantified by ImageLab™ software (v6.1).

### 2.12. 16S rRNA Sequencing

Fecal genomic DNA was isolated from murine specimens using the QIAamp DNA Mini Kit (Qiagen, Hilden, Germany). The V3-V4 hypervariable regions of bacterial 16S rRNA genes were amplified through PCR reactions (95 °C/3 min; 35 cycles: 95 °C/30 s, 55 °C/30 s, 72 °C/45 s; 72 °C/10 min) with primers 338F (5′-ACTCCTACGGGAGGCAGCA-3′) and 806R (5′-GGACTACHVGGGTWTCTAAT-3′). Amplified products were purified by AMPure XP beads (Beckman Coulter, USA) and quantified fluorometrically (Qubit 4.0, Thermo Fisher Scientific, Waltham, MA, USA). Sequencing libraries were prepared following the Illumina TruSeq Nano DNA LT protocol (Illumina, San Diego, CA, USA). Paired-end sequencing (2 × 250 bp) was performed on the Illumina NovaSeq 6000 platform. Raw reads were quality-filtered using FLASH v1.2.11 to generate high-fidelity merged tags. Operational Taxonomic Units (OTUs) were clustered at a 97% identity threshold using USEARCH v9.2.64 with the UPARSE-OTU algorithm, followed by taxonomic annotation against the SILVA 138 database. Alpha diversity was characterized by the observed species index, while beta diversity was assessed via principal coordinates analysis (PCoA). Microbial biomarkers were identified through LEfSe analysis (LDA score > 2.0, *p* < 0.05) using the online tool (http://huttenhower.sph.harvard.edu/galaxy/, accessed on 14 December 2023). Spearman rank correlations were computed using the R stats package (v4.2.1).

### 2.13. Quantification of Short-Chain Fatty Acid (SCFA)

SCFAs in mice intestinal content were quantified by gas chromatography–mass spectrometry (GC-MS, Agilent Technologies, Santa Clara, CA, USA). Fresh samples (50–100 mg) were homogenized with ice-cold PBS (1:4 *w*/*v*), acidified with 10% H_2_SO_4_, and spiked with 100 μL cyclohexanone internal standard (10 mg/L in methanol). After centrifugation (12,000× *g*, 10 min, 4 °C), take the supernatant for testing. Chromatographic separation employed a DB-WAX UI capillary column (30 m × 0.25 mm × 0.25 μm; Agilent) with the following parameters: carrier gas was high-purity helium (purity ≥ 99.999%) at a flow rate of 1.0 mL/min; inlet temperature 220 °C; injection volume of 1 μL; non-split injection; and solvent delay time of 2 min. MS conditions: electron bombardment ion (EI) source, ion temperature 230 °C, and interface temperature 220 °C. The data integration was conducted using MS quantitative analysis software, concentration calculations from standard curves, and quantification using internal standard methods. Quantitation was performed using MassHunter Workstation (v10.0). Analyte peaks were identified by retention time (±0.1 min) and characteristic ion ratios (±15% of standards). Data normalization incorporated internal standard correction.

### 2.14. Statistical Analysis

Blinding was applied during the stages of outcome assessment and data analysis. The researcher in charge of data collection and analysis had no knowledge of the group assignments, serving to avoid potential bias. All experiments in this study were conducted with three independent replications, and the results were expressed as mean ± SD. Data were analyzed and plotted using Excel 2019, SPSS 27, and GraphPad Prism 9 software. The significance of differences between groups was tested by one-way ANOVA (*p*-value < 0.05 indicated significant differences), and post hoc tests were used for multiple comparisons using Tukey’s test. Prior to conducting statistical analyses, the Shapiro–Wilk test and Levene test were used to assess data normality and variance homogeneity, respectively. In cases where these assumptions were violated, appropriate nonparametric tests were applied. Different letters in figures represent significant differences at *p* < 0.05.

## 3. Results

### 3.1. Biological Characteristics of Experimental Strains

**Figure A2:**
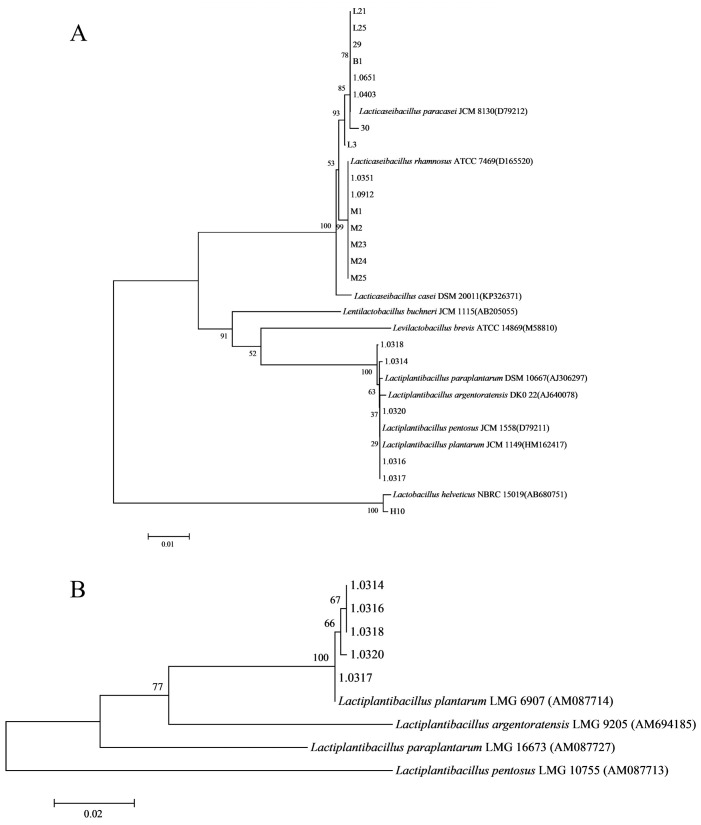
Biological identification of isolated strains. (**A**) Phylogenetic tree constructed based on 16S rRNA gene sequences. (**B**) Phylogenetic tree constructed based on the *pheS* gene of *Lactobacillus* spp.

The 21 lactic acid bacteria strains isolated and purified from yak milk were taxonomically identified as four species based on 16S rRNA and *pheS* gene sequencing (Figure A2A,B): *Lacticaseibacillus paracasei* (8 strains: 1.0403, 1.0651, 29, 30, L3, L21, L25, B1), *Lacticaseibacillus rhamnosus* (7 strains: 1.0351, 1.0912, M1, M2, M23, M24, M25), *Lactobacillus helveticus* (1 strain: H10), and *Lactiplantibacillus plantarum* (5 strains: 1.0314, 1.0316, 1.0317, 1.0318, 1.0320).

**Figure 1:**
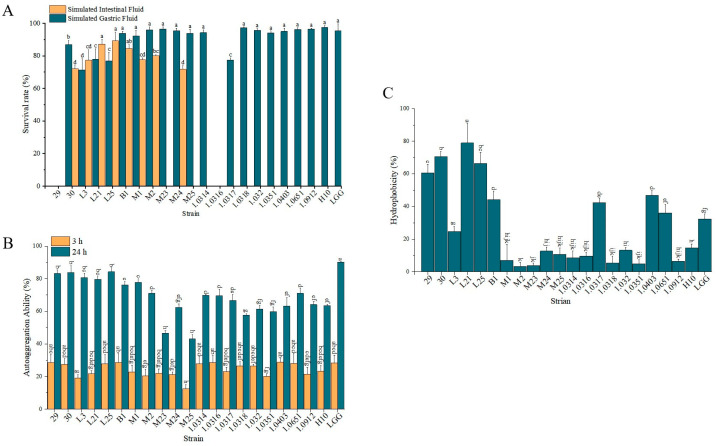
Evaluation of probiotic characteristics of 21 lactic acid bacterial strains. (**A**) Gastrointestinal tolerance assessed by survival rates under simulated gastric and intestinal conditions. (**B**) Auto-aggregation capacity assessed by the percentage of cell aggregation after 3 or 24 h of static incubation. (**C**) Cell surface hydrophobicity. Each experiment was replicated three times. The data represent the mean ± SD.

To screen for strains with excellent probiotic properties, the probiotic potential of 21 strains was assessed by simulated gastrointestinal tolerance, autoaggregation capacity, and hydrophobicity, with LGG as the positive control. These properties are necessary for the colonisation of the gut by live bacteria. As shown in Figure 1A, strains M23, M25, 1.0314, 1.0317, 1.0318, 1.0320, 1.0351, 1.0403, 1.0651, 1.0912, and H10 showed comparable survival in simulated gastric fluid, but did not survive in simulated intestinal fluid. Strains 29 and 1.0316 exhibited no tolerance in both simulated environments. Notably, strains B1, M1, M2, and M24 demonstrated gastric and intestinal survival rates comparable to LGG (*p* > 0.05). Despite reduced gastric tolerance, strains 30, L3, L21, and L25 displayed significantly higher intestinal survival than LGG (*p* < 0.05). Autoaggregation assays identified strains B1, 30, L21, and L25 with aggregation capacities similar to LGG (Figure 1B). Surface hydrophobicity results revealed strain L21 (78.86%) and B1 (44.17%) as the highest and lowest performers, respectively, both significantly higher than LGG (32.33%; *p* < 0.05, Figure 1C). These findings indicate that *Lacticaseibacillus paracasei* (hereafter referred to as *L. paracasei*) strains B1, 30, L21, and L25 have high potential for adherence to colonic epithelial cells and basal probiotic properties, which are suitable for further studies.

### 3.2. In Vitro Assessment of the Protective Effect of Probiotics on LPS-Induced Inflammation and Barrier Function in IEC-6 Cells

**Figure 2:**
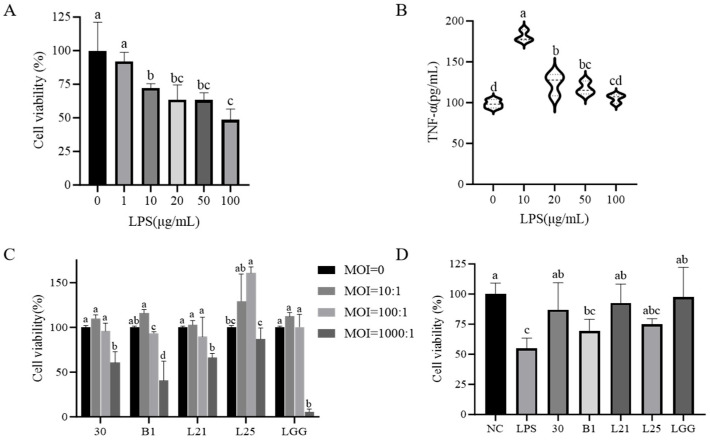
Bacterial strain effects on LPS-induced inflammatory responses in IEC-6 cells. (**A**) Impact of LPS concentration on IEC-6 cell viability. (**B**) Effect of LPS on TNF-α secretion by IEC-6 cells. (**C**) Bacterial dose-dependent effects on cell viability under varying multiplicity of infection. (**D**) Bacterial strains against LPS-induced cytotoxicity. Each experiment was replicated five times. The data represent the mean ± SD.

**Figure 3:**
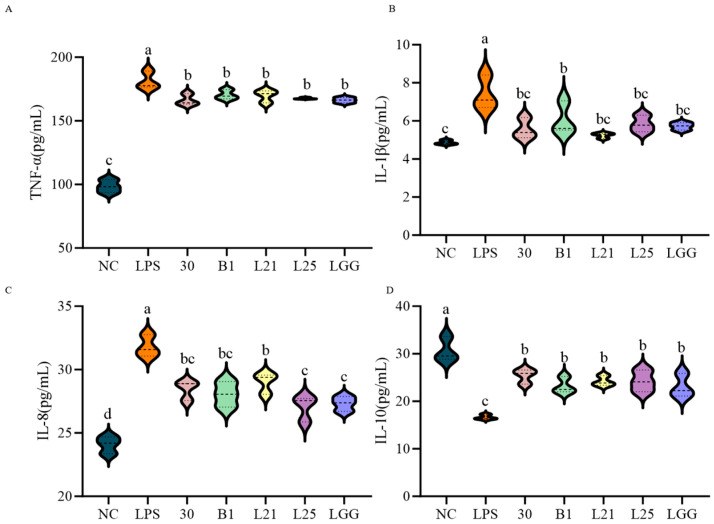
Effect of lactic acid bacteria on LPS-induced secretion of inflammatory factors by IEC-6 cells. (**A**) TNF-α. (**B**) IL-1β. (**C**) IL-8. (**D**) IL-10. NC group: blank control group; LPS group: model group. Each experiment was replicated three times.

In order to ensure the validity of the cell model, cytotoxicity assays were used to determine the optimal LPS concentration and multiplicity of infection (MOI) for bacterial-IEC-6 cell interactions. Compared to controls, LPS concentrations ≥10 μg/mL significantly reduced cell viability (*p* < 0.05) and increased TNF-α secretion (*p* < 0.05). Therefore, 10 μg/mL of LPS was used to establish the cellular inflammation model (Figure 2A,B). MOI results showed no significant cytotoxicity at a 10:1 ratio for all five strains (LGG, B1, 30, L21, L25), which confirmed that the 10:1 MOI ratio was appropriate for the model (Figure 2C). Subsequently, co-culture of LPS (10 μg/mL) with strains at MOI = 10:1 demonstrated differential effects on IEC-6 survival. Strains 30 and L21 significantly reduced cytotoxicity compared to the LPS group (*p* < 0.05) and achieved cell viability levels comparable to LGG (*p* > 0.05; Figure 2D). Analysis of inflammatory factors in the co-culture supernatants showed that LPS-treated cells exhibited increased levels of the pro-inflammatory cytokines TNF-α, IL-1β, and IL-8 (*p* < 0.05) and decreased levels of the anti-inflammatory cytokine IL-10 (*p* < 0.05) compared to controls (Figure 3A–D). But all strains tested significantly reversed these cytokine changes (*p* < 0.05), effectively restoring the inflammatory/anti-inflammatory balance (Figure 3A–D).

**Figure 4:**
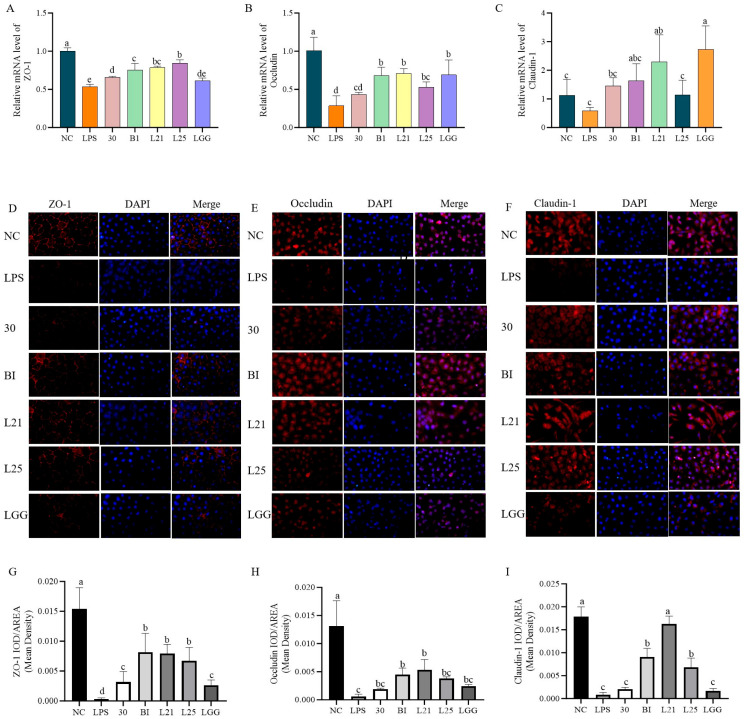
Modulation of tight junction protein expression in LPS-induced IEC-6 cells by lactic acid bacteria. (**A**) qRT-PCR analysis of mRNA levels of ZO-1. (**B**) qRT-PCR analysis of mRNA levels of occludin. (**C**) qRT-PCR analysis of mRNA levels of claudin-1. (**D**) Immunofluorescence localization of ZO-1. (**E**) Immunofluorescence localization of occludin. (**F**) Immunofluorescence localization of claudin-1. (**G**) Quantitative analysis of immunofluorescence intensity (mean optical density) for ZO-1. (**H**) Quantitative analysis of immunofluorescence intensity (mean optical density) for occludin. (**I**) Quantitative analysis of immunofluorescence intensity (mean optical density) for claudin-1. Proteins were green in IEC-6 cells, and nuclei were blue counterstained with DAPI. The data represent the mean ± SD.

Following this, the functional role of the strains in the barrier protection of LPS-induced IEC-6 cells was evaluated. Compared with normal controls, LPS treatment significantly reduced the mRNA relative expression of tight junction proteins ZO-1 and occludin (*p* < 0.05), whereas claudin-1 expression did not significantly decrease (*p* > 0.05, Figure 4A–C). Strains B1, L21, and L25 significantly upregulated the expression of ZO-1 and occludin (*p* < 0.05). In particular, *L. paracasei* *L21* exhibited the most pronounced restoration of occludin and claudin-1 levels, reaching values comparable to the LGG group (*p* > 0.05). Immunofluorescence localization revealed a distinct honeycomb-like distribution of ZO-1, occludin, and claudin-1 along cell membranes in untreated controls (red fluorescence encircling blue-stained nuclei; Figure 4D–F). The LPS treatment reduced the fluorescence intensity (Figure 4G–I). Following treatment with strains B1, L21, L25, and LGG, the membrane-associated fluorescence intensity was moderately increased compared to the LPS group. Quantitative analysis of fluorescence intensity indicated that strains B1 and L21 significantly enhanced the signal intensity of all three tight junction proteins in membrane-associated regions (Figure 4G–I, *p* < 0.05), suggesting a potential improvement in barrier function.

### 3.3. L. paracasei L21 and H-L21 Mitigate the Histopathology and Basic Indicators of DSS-Induced UC Mice

**Figure 5:**
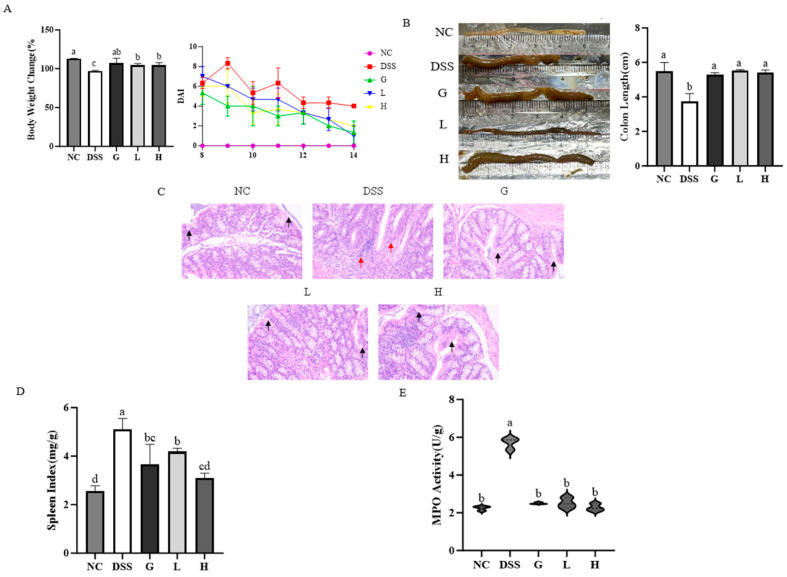
Experimental design and pathological/pathophysiological changes. (**A**) Body weight changes, and DAI score, a higher number indicates more severe colitis. (**B**) Colon length and macro image. (**C**) Colonic HE staining; black arrows indicate goblet cells and red arrows indicate inflammatory cells. (**D**) Spleen index of mice. (**E**) Activity of myeloperoxidase (MPO) in mouse colon tissue. The data represent the mean ± SD. The blank group is NC, DSS treatment group is DSS, mesalamine treatment group is G, *L. paracasei* *L21* treatment group is L, inactivation of *L. paracasei L21* treatment group is H.

*L. paracasei L21* showed excellent probiotic capacity and anti-inflammatory effects after in vitro screening of 21 candidate strains. To compare the ameliorative effects of *L. paracasei* *L21* probiotic and heat-inactivated *L. paracasei* *L21* (H-L21), both formulations were evaluated in a DSS-induced UC mice model. The results showed that compared to the NC group, DSS administration induced significant weight loss and disease activity index increase (*p* < 0.05; Figure 5A). Compared to the DSS group, both *L. paracasei* *L21* and H-L21 interventions restored body weight to baseline levels (*p* < 0.05) with results comparable to mesalazine (*p* > 0.05). In addition, the colon shortening (Figure 5B), mucosal damage (crypt atrophy, goblet cell loss, Figure 5C), and splenomegaly (Figure 5D) in DSS mice were significantly ameliorated by *L. paracasei* *L21* and H-L21 treatments. The colonic MPO activity, as a marker of neutrophil infiltration, was inhibited by *L. paracasei* *L21* and H-L21 to levels not significantly different from mesalazine (Figure 5E). Crucially, no statistically significant differences (*p* > 0.05) were observed between *L. paracasei* *L21* and H-L21 across most indicators (weight recovery, DAI, colon length, histopathology, MPO), suggesting both live and inactivated *L. paracasei* *L21* exert comparable effects in mitigating UC progression.

### 3.4. L. paracasei L21 and H-L21 Interventions Balance Inflammatory Dysregulation and Restore the Intestinal Barrier

**Figure 6:**
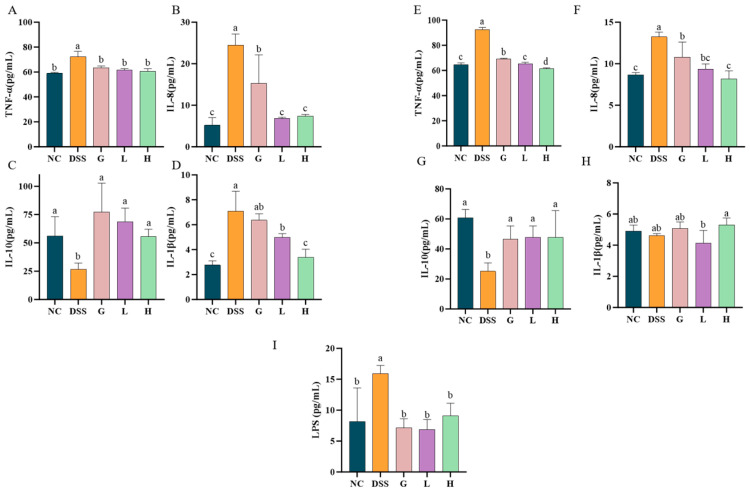
Levels of inflammatory factors in mouse colon tissue and serum. (**A**) Colon tissue levels of TNF-α. (**B**) Colon tissue levels of IL-8. (**C**) Colon tissue levels of IL-10. (**D**) Colon tissue levels of IL-1β. (**E**) Serum levels of TNF-α. (**F**) Serum levels of IL-8. (**G**) Serum levels of IL-10. (**H**) Serum levels of IL-1β. (**I**) LPS concentration in serum. The data represent the mean ± SD.

The imbalance between pro-inflammatory and anti-inflammatory factors is a common feature of IBD. Compared to the NC group, the DSS group exhibited significantly elevated levels of pro-inflammatory factors TNF-α and IL-8 in both colon tissues and serum (*p* < 0.05; Figure 6A,B,E,F). While changes in IL-1β levels in colon tissues were not pronounced (*p* > 0.05), its concentration in serum increased significantly (*p* < 0.05; Figure 6D,H). Additionally, a marked reduction in the anti-inflammatory cytokine IL-10 (*p* < 0.05) confirmed inflammatory dysregulation (Figure 6C,G). Both *L. paracasei* *L21* and H-L21 treatments effectively reduced TNF-α and IL-8 expression in local and systemic (*p* < 0.05), with the postbiotic group exhibiting greater IL-8 inhibitory efficacy. While IL-10 levels showed upward trends in treatment groups, these changes did not reach statistical significance (*p* > 0.05). Concurrently, serum LPS quantification revealed intestinal barrier disruption in DSS mice compared to group NC (*p* < 0.05; Figure 6I). *L. paracasei* *L21* and H-L21 significantly lowered serum LPS concentrations relative to the DSS group (*p* < 0.05), and were not significantly different from group G (*p* > 0.05).

**Figure 7:**
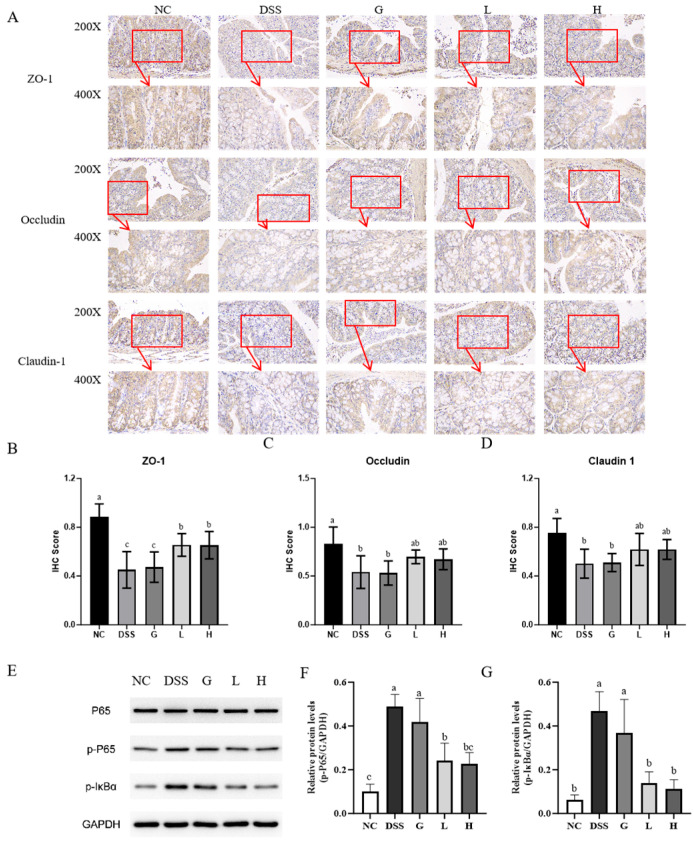
Expression of tight junction proteins and activation of signaling pathways in the intestinal barrier. (**A**) Immunohistochemical localization of tight junction protein in colon tissue and the 400× magnification view of the area inside the box. (**B**) IHC score of ZO-1. (**C**) IHC score of occludin. (**D**) IHC score of claudin-1. (**E**) Protein expression of NF-κB pathway-related gene. (**F**) Relative protein levels of *p*-P65. (**G**) Relative protein levels of *p*-IκBα. Different letters represent significant differences at the *p* < 0.05 level. The data represent the mean ± SD.

Based on the observed reduction in serum LPS, which indicated the recovery of the intestinal barrier, we further detected the colonic tight junction proteins by immunohistochemical analysis (Figure 7A). DSS mice exhibited significantly reduced ZO-1, occludin, and claudin-1 protein signal intensities compared to group NC, with corresponding reductions in IHC scores (*p* < 0.05; Figure 7B–D). *Lactobacillus paracasei* *L21* and H-L21 interventions did not significantly increase the IHC scores of 0ccludin and claudin-1 (*p* > 0.05), but particularly and significantly enhanced the signal intensity and IHC scores of the ZO-1 protein (*p* < 0.05), suggesting that it may play a key role in barrier or mucosal repair.

WB results revealed DSS-induced activation of the NF-κB pathway, evidenced by increased *p*-P65 and *p*-IκBα levels compared to group NC (*p* < 0.05; Figure 7E–G). However, treatment with *L. paracasei L21* and H-L21 reduced the phosphorylation of P65 and *p*-IκBα proteins, and were not significantly different from group G. These findings indicate that *L. paracasei L21* and H-L21 decrease local and systemic inflammation by inhibiting activation of the NF-κB pathway while restoring intestinal barrier integrity to limit LPS release.

### 3.5. L. paracasei L21 and H-L21 Alleviate DSS-Induced Colitis Through SCFA-Mediated Activation of AhR/HIF1α, Induction of IL-22 Production

**Figure 8:**
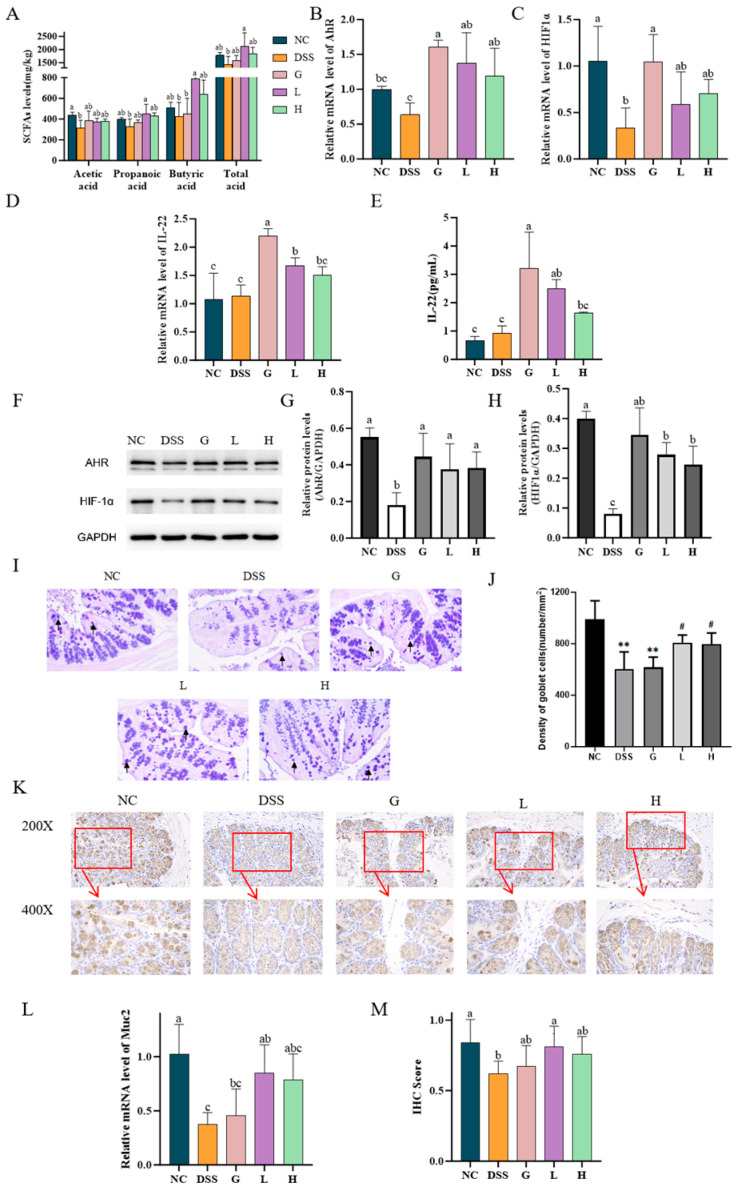
Content of SCFAs and expression of colitis-related signaling pathway proteins in the mice colon. (**A**) Content of SCFAs. (**B**) Relative mRNA expression levels of AhR. (**C**) Relative mRNA expression levels of HIF1α. (**D**) Relative mRNA expression of IL-22. (**E**) Expression of IL-22. (**F**) Expression of HIF1α and AhR proteins. (**G**) Relative protein levels of AhR. (**H**) Relative protein levels of HIF1α. (**I**) Colonic AB-PAS staining. (**J**) Number of goblet cells. (**K**) Immunohistochemical image of Muc2 and the 400× magnification view of the area inside the box. (**L**) Changes in relative mRNA expression of colonic Muc2. (**M**) IHC score of Muc2 protein. The data represent the mean ± SD. Note: ** indicates a highly significant difference between the two groups compared with the NC group (*p* < 0.01). # indicates a significant difference between the two groups compared with the DSS group (0.01 < *p* < 0.05).

SCFAs have been demonstrated to profoundly influence several physiological processes. DSS-induced colitis significantly reduced colonic SCFAs, including acetic acid, propionic acid, butyric acid, and total acids, compared to the NC group, with acetic acid exhibiting the most pronounced decrease (*p* < 0.05; Figure 8A). These reductions were associated with impaired intestinal barrier integrity and immune dysregulation. Intervention with *L. paracasei L21* and H-L21 restored SCFA production, particularly in the L group, which showed the highest increase (*p* < 0.05). To elucidate the link between SCFAs and mucosal repair, we analyzed key signaling pathways regulating intestinal immunity. Aryl hydrocarbon receptor (AhR) and hypoxia-inducible factor 1α (HIF1α) are critical regulators of IL-22 production and epithelial homeostasis. DSS induction significantly inhibited both mRNA and protein expression of AhR and HIF1α in colon tissues compared to NC (*p* < 0.05; Figure 8B,C,F–H). *L. paracasei* *L21* and H-L21 significantly reversed AhR and HIF1α expression (*p* < 0.05), with AhR levels in the L group similar to those of NC (*p* > 0.05). Accordingly, IL-22 mRNA expression and protein concentration were highly increased in group L and H compared to DSS (*p* < 0.05; Figure 8D,E).

In order to ascertain the restoration effect of *L. paracasei* *L21* and H-L21 on intestinal barrier damage, AB-PAS staining was performed. The results revealed that mucin was abundant in the goblet cells of the NC group, mainly distributed on the surface of colon epithelial cells. In contrast, mice in the DSS group appeared to have goblet cell depletion, abnormal MUC2 secretion, and downregulated MUC2 mRNA and protein expression levels (*p* < 0.05; Figure 8I,J,L). Both *L. paracasei* *L21* and H-L21restored goblet cell numbers and promoted MUC2 secretion, with *L. paracasei* *L21* being more significant than H-L21 (*p* < 0.05). Immunohistochemical analysis further confirmed that L groups significantly ameliorated DSS-induced MUC2 protein loss (*p* < 0.05). However, the difference in IHC scores between the H group and the DSS group was not significant, which is consistent with the results of Muc2 protein mRNA expression. In summary, these findings demonstrate that *L. paracasei* *L21* and its postbiotic mitigate colitis by enhancing SCFA-mediated activation of AhR/HIF1α signaling, thereby promoting IL-22-dependent mucin synthesis and goblet cell regeneration.

### 3.6. Lactobacillus paracasei L21 and H-L21 Reverse DSS-Induced Gut Microbiota Imbalance in UC Mice

**Figure 9:**
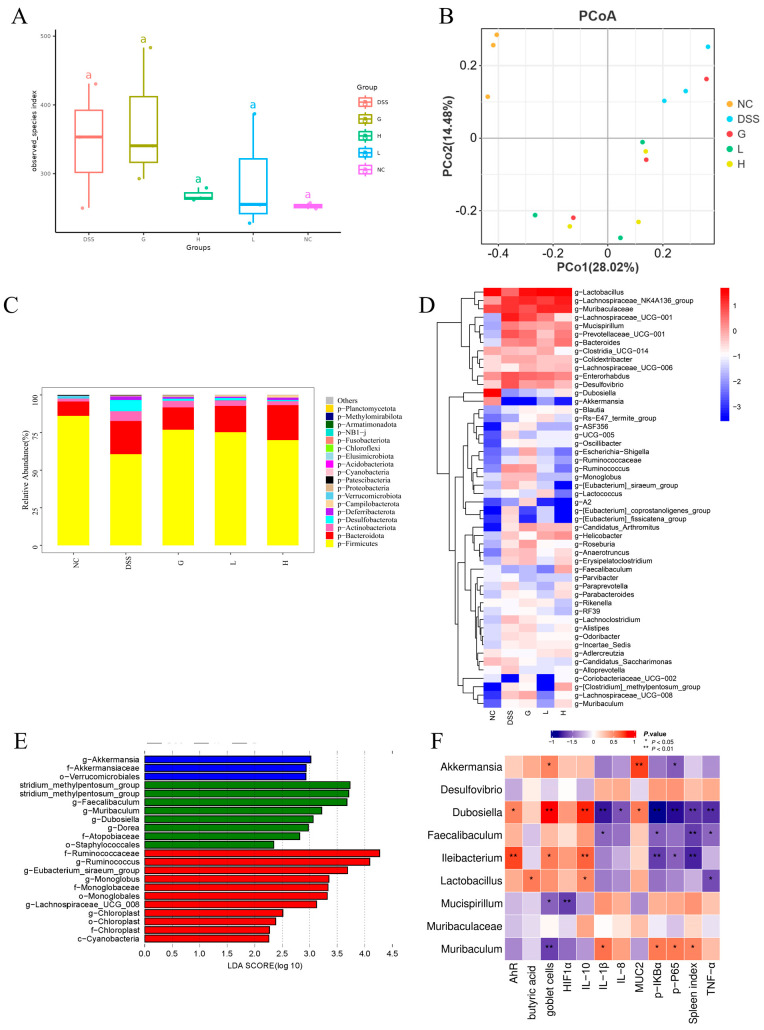
16S rDNA results of mouse colon contents. (**A**) The observed species Index. (**B**) Principal coordinate analysis (PCoA) plot. (**C**) The composition of gut microbiota at phylum level. (**D**) The composition of gut microbiota at genus level. (**E**) LEfSe analysis, LDA > 2.0. (**F**) Heatmap of Spearman correlation analysis. Red represents positive correlation, purple represents negative correlation, and the darker the colour, the stronger the correlation. The darker the color, the stronger the correlation. * Denotes significant difference (*p* < 0.05), ** Denotes highly significant difference (*p* <0.01), *n* = 6. The data represent the mean ± SD.

Current studies have found that the pathogenesis and progression of colitis are associated with characteristic changes in the structure of the gut microbiota. Therefore, this study evaluated the modulation of *L. paracasei L21* and H-L21 on DSS-induced gut microbiota in UC mice. Analysis of alpha-diversity revealed an increased species index in the DSS group compared to the NC group (*p* > 0.05), indicating a disruption of the microbiota (Figure 9A). While the L and H groups showed restored indices approaching NC levels, suggesting partial recovery of microbial diversity. Beta diversity demonstrated distinct clustering among groups. The samples from the DSS group were primarily concentrated in the upper right of the coordinate axis, with a notable degree of independence (*p* > 0.05; Figure 9B). Although the L, H, and G groups were a certain distance from the NC group, the sample points of the three groups generally overlapped, and the microbial structure was similar.

At the phylum level, compared to the NC group, DSS induction significantly reduced Firmicutes and increased Bacteroidota, Actinobacteriota, and Desulfobacterota. Both *L. paracasei L21* and H-L21 interventions reversed these changes, restoring Firmicutes abundance, with the L group showing great efficacy in reducing Bacteroidota (*p* < 0.05; Figure 9C). At the genus level (Figure 9D), compared with the NC group, the relative abundance of 12 main genera increased in the DSS group (*Lachnospiraceae_NK4A136_group*, *Muribaculaceae*, *Lachnospiraceae_UCG-001*, *Enterorhabdus*, *Prevotellaceae_UCG-001*, *Desulfovibrio*, *Bacteroides*, *Mucispirillum*, *Helicobacter*, *Colidextribacter*, *Ruminococcus*, and *Candidatus_Arthromitus*), and the relative abundance of three genera decreased (*Lactobacillus*, *Dubosiella*, and *Clostridia_UCG-014*). Compared to the DSS group, the relative abundance of eight genera in the L and H groups returned to near NC levels, including six genera whose relative abundance increased significantly after DSS induction (*Lachnospiraceae_UCG-001*, *Enterorhabdus*, *Prevotellaceae_UCG-001*, *Desulfovibrio*, *Mucispirillum*, and *Ruminococcus*) and two reduced genera (*Lactobacillus* and *Dubosiella*). In addition, distinct from the G and H groups, the L groups could significantly increase the relative abundance of *Akkermansia* (*p* < 0.05).

LEfSe analysis identified *Akkermansia* as a marker genus in the L group (LDA > 3.0), while the H group featured *Clostridium_methylpentosum_group*, *Faecalibaculum*, and *Dubosiella* (LDA > 3.5, Figure 9E). More importantly, correlation analysis revealed that the goblet cell numbers and MUC2 expression were significantly and positively correlated with *Akkermansia* and *Dubosiella* (*p* < 0.05; Figure 9F). Besides, *Dubosiella* is also strongly associated with inflammatory factors (IL-8, IL-10, IL-1β, and TNF-α) and NF-κB/AHR/HIF-1α signaling pathway (*p* < 0.05). These findings indicate that *L. paracasei* *L21* and H-L21 may ameliorate DSS-induced dysbiosis by restoring core taxa and modulating microbiota–immune interactions.

## 4. Discussion

Recent evidence highlights the critical role of gut microbiota in modulating host immunity and suppressing intestinal inflammation, driving increased demand for probiotic-enriched functional foods [27]. Research showed that members of the lactic acid bacteria exhibit multifaceted probiotic properties, including microbiota modulation, pathogen exclusion, and immunoregulatory effects [28]. In this study, we isolated 21 lactic acid bacterial strains from traditional fermented dairy products in China. While 16S rRNA sequencing remains a standard taxonomic tool, its limited resolution for closely related species necessitated complementary phylogenetic analysis using housekeeping genes to ensure accurate classification. The results showed that the 21 isolated lactic acid bacteria were classified as *Lacticaseibacillus paracasei*, *Lactiplantibacillus plantarum*, *Lacticaseibacillus rhamnosus*, and *Lactobacillus helveticus*, respectively (Figure A1). Survival through gastrointestinal (GI) stressors, including gastric acid, proteolytic enzymes, and bile salts, is a prerequisite for probiotic efficacy [29]. LGG, a well-known commercial probiotic with good gastrointestinal tolerance and colonization ability, is often used as a positive control [30]. Notably, our study found that eight strains (*L. paracasei* 30, L3, L21, L25, B1; *L. rhamnosus* M1, M2, M24) demonstrated superior GI tolerance comparable to the reference strain LGG (Figure 1). Probiotic adhesion to intestinal epithelia, mediated by cell surface hydrophobicity and autoaggregation capacity, is crucial for colonization and pathogen exclusion [31]. We used xylene and ethyl acetate affinity to test the hydrophobicity of all strains. Compared to LGG, *Lactobacillus* 30, L21, L25, B1, and 1.0651 had higher cell—surface hydrophobicity. Furthermore, all isolates displayed strong autoaggregation, a phenotype associated with biofilm formation and competitive exclusion of enteropathogens [32]. Combined, these findings suggest strains 30, L21, L25, and B1 in this study possess exceptional probiotic potential for intestinal persistence (Figure 1).

Our study next used an LPS-induced IEC-6 enterocyte inflammation model with LGG as a positive control, and evaluated in vitro anti-inflammatory capacity of four *L. paracasei* strains (30, L21, L25, B1) with great probiotic potential. The IEC-6 cell line is extensively utilized as an in vitro model for intestinal inflammation studies owing to its capacity to replicate the barrier functionality of intestinal epithelial cells [33,34]. Studies have shown that certain probiotics can be associated with a range of host immunomodulatory activities, including downregulation of pro-inflammatory gene expression and cytokine production [35]. LPS, a key component of Gram-negative bacterial membranes, triggers systemic inflammation by stimulating TNF-α production. TNF-α is a master regulator of proinflammatory signaling that modulates cell proliferation, apoptosis, and immune dysregulation [36]. IL-10 exerts immunomodulatory effects by suppressing proinflammatory cytokine cascades, positioning it as a therapeutic target for inflammatory disorders [37,38]. As expected, all test strains in our study significantly attenuated proinflammatory mediators while elevating IL-10 levels (*p* < 0.05). Similar effects of *L. paracasei* may predict the presence of conserved anti-inflammatory mechanisms in this subspecies. Especially strain L21 exhibited unique barrier-reinforcing properties through upregulated expression of tight junction proteins (ZO-1, occludin, claudin-1), thereby reducing paracellular permeability. This is a critical defense against endotoxin translocation [39]. These findings align with Dai et al.’s report on probiotic-mediated barrier restoration in LPS-stimulated IEC-6 cells [40], yet contrast with Niu et al.’s observation of IL-8 elevation by *Bifidobacterium* H4-2 under similar conditions [41]. This divergence highlights strain-specific immunomodulatory, and underlines the necessity for individualized probiotic screening.

The increasing use of probiotics in gut health management is supported by their ability to modulate immune responses and alleviate intestinal inflammation [27]. However, concerns remain about the safety of probiotics, particularly in immunocompromised patients and those with gastrointestinal disorders. Therefore, it is necessary to explore alternatives, such as postbiotics. Postbiotics offer inherent safety advantages in such patient groups. Since there are few studies directly comparing the effects of probiotics and their postbiotics, elucidating their mechanisms under normal physiological conditions is crucial for the development of effective dietary supplements for UC. Our study systematically compares the effects of live *L. paracasei* *L21* and its heat-inactivated postbiotic in a mouse colitis model, revealing that both forms significantly alleviate disease severity. The 2.5% DSS-induced colitis model, validated for its clinical relevance to human UC [42], successfully replicated pathological features including goblet cell depletion, epithelial disruption, and neutrophil infiltration. Histological analysis confirmed that *L. paracasei* *L21* and postbiotic interventions restored goblet cell density, while reducing inflammatory cell infiltration. These findings illustrate the ability of *L. paracasei* *L21* to preserve mucosal tissue regardless of bacterial viability. This is a critical advantage for clinical applications in susceptible population.

Enteritis frequently leads to changes in the permeability of the intestinal epithelium. The intestinal epithelium possesses a barrier function, which needs the presence of a continuous layer of cells and the preservation of the integrity of the paracellular space connection between tightly sealed epithelial cells [43]. In this study, we found that *L. paracasei* *L21* and H-L21, in particular, significantly increased the signal intensity and IHC score of ZO-1. ZO-1 is primarily responsible for connecting transmembrane proteins to the cytoskeleton and serves as a key structure for signal transduction [44]. Kuo et al. demonstrated that mucosal repair is severely compromised in ZO-1 knockout mice, suggesting that ZO-1 is critical for mucosal healing [45]. Thus, the effective restoration of ZO-1 by *L. paracasei* *L21* and H-L21 likely underlies the alleviation of mucosal damage observed in our model. Furthermore, our findings are consistent with those reported by Ahmad et al., who showed that *Lactobacillus paracasei* BNCC345679 restores goblet cell function and enhances the protective properties of the mucin layer by upregulating MUC2 and ZO-1 expression [46]. The upregulation of MUC2, a key mucin that maintains the protective mucus layer, suggests a double reinforcement of the intestinal barrier by *L. paracasei* *L21* and H-L21 through both ZO-1 and mucin [47]. The observed reduction in systemic LPS levels further supports restored barrier function, likely limiting bacterial translocation and subsequent inflammation [48].

MPO can facilitate the production of potent reactive oxygen species (ROS), which in turn sustain inflammation and lead to tissue damage in individuals with enteritis [49]. Studies have found that administering *Bifidobacterium longum* as a probiotic to mice with DSS-induced UC has decreased MPO activity and significantly improved colonic symptoms [50]. Similar to *Bifidobacterium longum*, our study also found that *L. paracasei* *L21* and its postbiotics effectively decreased the activity of MPO in the colon tissue of DSS-induced UC mice. They also considerably reduced the levels of pro-inflammatory cytokines TNF-α, IL-8, and IL-1β. To prevent the excessive release of inflammatory cytokines, it is necessary to inhibit the amplification of the inflammatory cascade by stimulating the buildup and activation of leukocytes [51]. Additionally, some *peptic lactobacilli* have been discovered to possess inhibitory properties against pro-inflammatory cytokines [43]. IL-10 was a prototypical anti-inflammatory cytokine that positively affected the immune response of the intestinal mucosa. Studies have shown that increasing the concentration of IL-10 positively impacted reducing inflammation caused by DSS in mice with UC [52].

The NF-κB protein typically exists in a stable state when bound to IκBα. Upon stimulation by exogenous stimuli, such as bacteria, the cellular complex begins degradation by phosphorylation and proteolysis. This degradation process facilitates the translocation of NF-κB to the nucleus, hence regulating the release of inflammatory cytokines [41]. Thus, the elevated levels of *p*-IκBα and *p*-p65, crucial components in the NF-κB signaling pathway, indicate the activation of the NF-κB signaling pathway, leading to subsequent inflammatory reactions. *L. paracasei* *L21* and its postbiotics treatments could prevent the activation of the NF-κB signaling pathway, reducing the intestinal inflammatory response in UC mice. Probiotics were also commonly observed to have anti-inflammatory effects by preventing the NF-κB signaling pathway [53].

In a healthy state, the gut flora maintains a certain homeostasis in the body. However, when the normal relationship between the host and microbes is disturbed, it leads to ecological imbalance and diseases in the gut [46]. Presently, it is widely acknowledged that the intestinal microbiota plays a crucial role in the recurrence and development of UC [54]. This imbalance can lead to the growth of harmful microorganisms in the intestines, causing damage to the intestinal barrier and making it easier for germs to move from the intestines to other parts of the body [55]. Some research indicated that living probiotics have a more potent immunostimulatory impact than inactivated probiotics [56]. Similar conclusions were widely supported in the study of *Loigolactobacillus coryniformis NA-3* [57] and *Bifidobacterium longum* subsp. *longum* CCM 7952 [58]. Nevertheless, it remains uncertain if the drivers stimulated by the postbiotics and probiotic therapies were consistent. Consequently, we analyzed the gut microbiota of mice following various treatments and aimed to investigate if there are specific biomarker species in the intestinal tract of mice with UC after receiving postbiotics therapies that differ from those of probiotic interventions. The high-throughput sequencing results of this work demonstrated that DSS causes disturbances in the intestinal flora of mice. Although there was no statistically significant difference in alpha diversity across the groups in this study (*p* > 0.05), the intervention of *L. paracasei* *L21* and its postbiotics could restore this alteration. The findings demonstrated that the therapy effectively inhibited the disruption of the intestinal microbial composition induced by DSS and reinstated the diversity and composition of the gut microbiota. Similarly, Xu, et al. observed that although the alpha diversity of the gut microbiota was not affected in colitis mice, treatment with a single strain of probiotic and a combination of probiotic strains restored the composition and structure of the host gut microbiota [59].

DSS-induced colitis led to a disruption in the structure of the intestinal flora in mice, characterized by a reduction in Firmicutes and an elevation in Bacteroidota at the phylum. Specifically, there was a significant decrease (*p* < 0.05) in the relative abundance of *Lactobacillus* and *Dubosiella* belonging to Firmicutes and a significant increase in the relative abundance of *Bacteroides*, *Muribaculaceae*, and *Prevotellaceae_UCG-001* belonging to Bacteroidota (*p* < 0.05). *L. paracasei* *L21* and its postbiotic treatment effectively corrected the imbalance of Firmicutes and Bacteroidota and restored the microbial structure. Additional research on the use of probiotics to improve colitis has also demonstrated that therapies involving *L. paracasei* [60], *Lactobacillus ruminis* [61], and *Bifidobacterium* [53] effectively counteract the reduction in Firmicutes and the rise in Bacteroidota in the gut microbiota of mice with DSS-induced colitis [62]. These results further support the potential that *L. paracasei* *L21* and its postbiotics may mediate microbial mitigation of UC. Following the use of *L. paracasei* *L21* and its postbiotic, there was a notable increase in the presence of advantageous intestinal symbiotic bacterial taxa, including *Lactobacillus*, *Dubosiella*, *Muribaculaceae*, *Akkermansia*, and *Candidatus_Arthromitus*. Most of them are members of the *Firmicutes* phylum and were widely recognized as probiotic genera [63]. These genera play a crucial role in regulating the composition of the human intestinal microbiota and promoting intestinal microecological balance. *Lactobacillus* has the potential to reduce the clinical symptoms of UC and help maintain a healthy balance in the intestines [64], and *Lactobacillus* is an important butyric acid producer [65]. In this work, the correlation analysis revealed a noteworthy positive association between the quantity of butyric acid and the presence of *Lactobacillus* in the colon of mice. Butyric acid is a significant SCFA that is synthesized by the microorganisms in the intestines to help avoid colitis [66].

*Dubosiella* shows the potential to decrease inflammation in the intestines and enhance the integrity of the intestinal mucosal barrier. The preventive efficacy of *Dubosiella* is attributed to its metabolites. *Dubosiella* can influence the balance of the immune system in the colon by producing SCFAs (particularly propionic acid and lysine) and activating metabolic pathways connected to the aryl hydrocarbon receptor in dendritic cells. By influencing interconnected metabolic pathways, *Dubosiella* can regulate the immune system and prevent inflammation in the colon, improving damage to the mucosal barrier [67]. The current results suggested that the increased concentrations of anti-inflammatory factor and down-regulation of pro-inflammatory factors, in group H, might be attributed to the influence of *Dubosiella* (Figure 9F). This study discovered a positive relationship between the AhR-related pathway and *Dubosiella*, suggesting that the AhR pathway can be a promising target for treating inflammatory bowel disease. This finding also adds to the existing evidence supporting the role of *Dubosiella* in influencing the AhR pathway. The LEfSe and correlation analysis indicated that *Dubosiella* was the primary species responsible for the observed differences between the postbiotics-treated group and the other groups. Our results showed that the relative abundance of *Dubosiella* was 0.22% in group H, whereas in group L it was 0.06%. *Dubosiella* also shows a significant correlation with various cytokines and signaling pathways, which implies that an increase in *Dubosiella* may potentially inhibit the NF-κB signaling pathway and reduce the inflammatory response in the intestines [68]. Meanwhile, *Dubosiella* positively regulates the release of the anti-inflammatory cytokine IL-10. Studies have demonstrated that up-regulating the expression of IL-10 could effectively mitigate DSS-induced colitis. This is achieved through synergistic regulation of the gut microbiota and restoration of intestinal barrier integrity [69]. Overall, in addition to protecting the intestinal barrier, *Dubosiella*, as a marker microbe of the postbiotic, also drives immune regulation in UC mice.

*Akkermansia,* a member of the Verrucomicrobia phylum, has the unique ability to degrade mucin glycoproteins while stimulating mucus production, thereby rendering a protective role in intestinal epithelial injury [70]. *Akkermansia* has the potential to degrade mucin to produce SCFA, which is the energy source for mucin synthesis and secretion by host epithelial cells, in particular, propionic acid [71]. In fact, our findings support this hypothesis and demonstrate that *Akkermansia* was a marker in the *L. paracasei* *L21* intervention group and correlation analyses showed a significant correlation with MUC2 and goblet cells levels. In short, our data provide evidence that the *L. paracasei* *L21* intervention group more predominantly increased SCFA through *Akkermansia* regulation of mucin and goblet cells levels to alleviate colonic inflammation in mice.

*Candidatus Arthromitus* is a recently established bacterial classification. *Candidatus Arthromitus*, a type of bacteria that lives in the intestines of animals, has been observed to mostly enter the mucus layer of the gut without attacking the cells that line the intestine. It triggers the immune system of the host by boosting T-cell responses [72]. *Candidatus Arthromitus* levels generally decline as the induction period increases in mice models induced by DSS [73]. This aligns with the findings of our investigation, which observed a decrease in the relative abundance of *Candidatus Arthromitus* in the DSS group. *Muribaculaceae* can promote the growth of beneficial microorganisms in the intestine. Research has demonstrated that the presence of *Muribaculaceae* in the gut can increase telomerase activity and alleviate inflammation in the intestines of older mice [74]. The increased abundance of *Muribaculaceae* might enhance the intestinal milieu, boost the production of SCFAs, and facilitate the suppression of colon lesions by inhibiting the processes associated with butyrate Salt synthesis [75]. The isolation and cultivation of *Muribaculaceae* in vitro has been a slow process. It is anticipated that *Muribaculaceae* will be used to treat inflammatory bowel disease in the future [76]. *Mucispirillum* is commonly recognized as a harmful microbe [77] and is associated with the development of IBD [10]. The increase in the relative abundance of *Mucispirillum* in the DSS group may have resulted in the inhibition of HIF1α expression and reduced the number of goblet cells, thus contributing to the development of colitis. Furthermore, *Desulfovibrio*, which is markedly increased in the intestines of colitis model mice, has been confirmed to facilitate the onset and progression of IBD through the production of hydrogen sulfide and acetate [78]. The above analysis showed that the intervention treatment of *L. paracasei* *L21* and its postbiotics could improve the intestinal flora structure of colitis model mice, reduce the level of pathogenic bacteria in the host intestine, and restore beneficial effects. The dominant position of beneficial microorganisms thus plays a positive role in alleviating colitis in mice.

The gut microbiota metabolizes carbohydrates through fermentation, producing SCFAs, which exert a protective influence on the cells that line the intestines by either activating or inhibiting the generation of inflammatory cytokines. Additionally, they can either hinder or promote the movement and recruitment of immune cells [79]. However, the basic mechanism by which SCFAs influence inflammatory processes has not yet been further understood. A study discovered that propionic acid had the most significant impact in reducing inflammation when probiotics were added to animals with DSS-induced UC [80]. Nevertheless, the specific objective of SCFAs has yet to be identified. AhR is an essential regulator of intestinal immunity and a promising therapeutic target for IBD. Hou et al. have shown that *Lactobacillus reuteri* D8 has the ability to protect the intestinal barrier and increase the growth of intestinal epithelial cells [81]. This is primarily achieved by enhancing the expression of AhR, which promotes the production of IL-22 by lamina propria lymphocytes [82]. The study discovered that the intervention of *L. paracasei* *L21* resulted in a significant increase in the content of SCFAs and the levels of AhR and HIF1α in the colon of mice with DSS-induced colitis, leading to an upregulation of IL-22 production. IL-22 activates the IL-22-STAT3 signaling pathway in intestinal epithelial cells by inducing STAT3 phosphorylation (pSTAT3). This activation contributes to intestinal immunity and helps maintain intestinal homeostasis, particularly in cases of chemically induced colitis [83].

**Figure 10:**
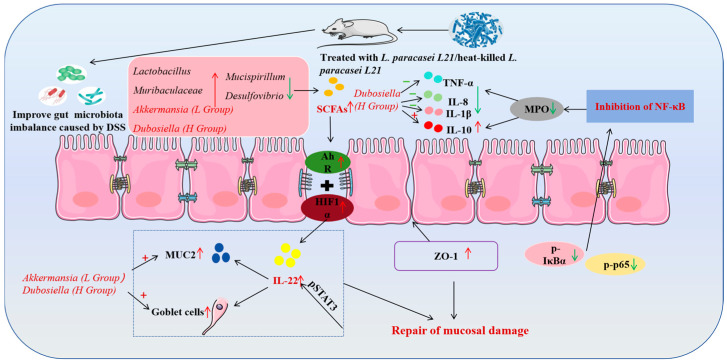
Mechanism of *L. paracasei* *L21* and its postbiotics in the treatment of colitis induced by DSS in mice. Red arrows indicate increased abundance in the treated group and green arrows indicate decreased. + for positive correlation, − for negative correlation.

In summary, as shown in Figure 10, *L. paracasei* *L21* and its postbiotics effectively improved the intestinal flora’s structural imbalance in mice with colitis. This increased the abundance of the beneficial probiotic organisms *Dubosiella*, *Lactobacillus*, and *Muribaculaceae* while reducing the abundance of potentially harmful bacteria such as *Desulfovibrio* and *Mucispirillum*. *Dubosiella* plays a significant role in reducing inflammation through postbiotic components. The higher level of SCFAs led to a considerable increase in the levels of IL-22 by stimulating the expression of AhR and HIF1α. IL-22 protected the digestive tract from inflammation by increasing the number of goblet cells and MUC2 production. Meanwhile, upregulation of the tight junction protein ZO-1 promoted recovery from mucosal injury and reduced intestinal permeability. Furthermore, the administration of *L. paracasei* *L21* and its postbiotics intervention decreased blood inflammation levels, improved intestinal tissue damage, and intestinal inflammation symptoms in mice by inhibiting the NF-κB signaling pathway.

## 5. Conclusions

This study identified *L. paracasei* *L21* as a potential probiotic with UC- UC-alleviating properties. Through a combination of in vitro and in vivo models, *L. paracasei* *L21* exhibited anti-inflammatory activity by balancing dysregulated cytokines and enhancing the intestinal barrier. *L. paracasei* *L21* and its heat-inactivated postbiotic mitigated DSS colitis similarly attenuated DSS-colitis by modulating the NF-κB and HIF1α/AhR-IL-22-MUC2 axes. However, *L. paracasei* *L21* and its postbiotic restored the core gut microbiota in different ways. *L. paracasei* *L21* enriched *Akkermansi*, while the marker species of the postbiotic is *Dubosiella*. This study used a small sample size of mice to establish a DSS-induced acute colitis model, which cannot fully simulate the complex pathology of human IBD. Secondly, the SPF-grade breeding environment where the mice were housed differs from the diverse living environments of humans, which may limit the generalizability of the results. Future research should include large-scale cohort studies and better alternative models. Nonetheless, the comparable efficacy of live and heat-inactivated *L. paracasei* *L21* addresses potential safety concerns in immunocompromised populations, providing a scientific rationale for the development of *L. paracasei* L21-based probiotic/postbiotic formulations, and it also contributes to the exploration of next-generation UC therapeutics.

## Data Availability

This study was not pre-registered in a publicly accessible protocol repository, as is common in preclinical animal research. Future studies will aim to follow standardized protocol registration practices. The data relevant to the article are available from the corresponding author upon request.

## Appendix A

**Table A1:** Primer sequence of PCR amplification.

Purpose	Gene Name	Primer Sequence (5′-3′)
Primer for 16SrRNA gene amplification	*16S rRNA* (27F)	AGAGTTTGATCCTGGCTCAG
	*16S rRNA* (1492R)	CTACGGCTACCTTGTTACGA
Primers for housekeeping gene amplification	*pheS* (F)	CAYCCNGCHCGYGAYATGC
	*pheS* (R)	GGRTGRACCATVCCNGCHCC
Sequences of RT-qPCR (intestinal barrier, cells)	*ZO-1*	GCCAAGCCAGTCCATTCTCAGAG TCCATAGCATCATTTCGGGTTTCC
	*Occludin*	CAACGGCAAAGTGAATGGCAAGAG TCATCCACGGACAAGGTCAGAGG
	*Claudin-1*	CTTCTGGGTTTCATCCTGGCTTCG CCTGAGCAGTCACGATGTTGTCC
Sequences of RT-qPCR (intestinal barrier, animals)	*ZO-1*	CTTCTCTTGCTGGCCCTAAAC TGGCTTCACTTGAGGTTTCTG
	*Occludin*	CACACTTGCTTGGGACAGAG TAGCCATAGCCTCCATAGCC
	*Claudin-1*	TCTACGAGGGACTGTGGATG TCAGATTCAGCTAGGAGTCG
	*MUC2*	TGGTCCAGGGTTTCTTACTCC TGATGAGGTGGCAGACAGGAGAC
IL-22 and related signaling pathways	*IL-22*	AACAGCAGGTCCAGTTCCCC GCTCCTGTCACA TCAGCGGT
	*AhR*	TTGGTTGTGATGCCAAAGGGC CATGCGGATGTGGGATTCTGC
	*HIF1α*	AGCTTCTGTTA TGAGGCTCACC TGACTTGATGTTCATCGTCCTC
Reference gene (cells)	*β*-*actin*	GTTGACATCCGTAAAGACCT CCACCAATCCACACAGAGTA
Reference gene (animals)	*GAPDH*	TCAAGAAGGTGGTGAAGCAG AAGGTGGAAGAGTGGGAGTTG

**Table A2:** Nutrient composition of AIN-93 feed.

Ingredients	Energy Content
Protein (Casein)	17.8%
Carbohydrates (dextrin, sucrose, cornstarch, cellulose)	64.3%
Fats (soybean oil)	7%
Water	6.6%
Mineral (ash content)	4.17%
Vitamin and amino acid complexes (Methionine, choline, vitamins, etc.)	0.13%
Total	100%
Energy (kcal/kg)	3766

**Table A3:** Evaluation standard of DAI.

Score	Weight Loss Rate (%)	Faecal Patterns	Occult Blood/Hematochezia
0	0	Normal	Normal feces
1	1−5	−	−
2	5−10	Loose but formed feces	Feces with a small amount of blood
3	10−15	−	−
4	>15	Diarrhea	Feces with a large amount of blood

**Table A4:** Antibody Information.

Purpose	Primary/Secondary Antibodies	Antibody Name	Dilution Ratio	Manufacturers
Immunofluorescence	Primary antibodies	ZO-1	1:1000	Proteintech Group, Inc (Wuhan, China)
	Primary antibodies	Occludin	1:500	Proteintech Group, Inc (Wuhan, China)
	Primary antibodies	Claudin1	1:200	Affinity Biosciences (Cincinnati, OH, USA)
	Secondary antibodies	HRP-labelled rabbit anti-goat	1:400	SeraCare (Milford, MA, USA)
	Secondary antibodies	HRP labelled goat anti-rabbit	1:400	SeraCare (Milford, MA, USA)
	Secondary antibodies	HRP labelled goat anti-mouse	1:400	SeraCare (Milford, MA, USA)
	Secondary antibodies	HRP labelled goat anti-rat	1:200	SeraCare (Milford, MA, USA)
Immunohistochemistry	Primary antibodies	ZO-1	1:200	Proteintech Group, Inc (Wuhan, China)
	Primary antibodies	Occludin	1:400	Proteintech Group, Inc (Wuhan, China)
	Primary antibodies	Claudin1	1:100	Affinity Biosciences (Cincinnati, OH, USA)
	Primary antibodies	MUC2	1:1000	Guge Biological Technology (Wuhan, China)
	Secondary antibodies	HRP-labelled rabbit anti-goat	1:100	SeraCare (Milford, MA, USA)
	Secondary antibodies	HRP-labelled goat anti-rabbit	1:500	SeraCare (Milford, MA, USA)
	Secondary antibodies	HRP-labelled goat anti-mouse	1:500	SeraCare (Milford, MA, USA)
	Secondary antibodies	HRP-labelled goat anti-rat	1:500	SeraCare (Milford, MA, USA)
	Secondary antibodies	HRP-labelled goat anti-rabbit-mouse universal secondary antibody	1:1	DAKO (Copenhagen, Denmark)
Western Blot	Primary antibodies	GAPDH	1:10000	Proteintech (Chicago, IL, USA)
	Primary antibodies	AHR	1:5000	Proteintech (Chicago, IL, USA)
	Primary antibodies	HIF-1α	1:2000	Bioss (Beijing, China)
	Primary antibodies	P65	1:1000	ZenBioScience (Chengdu, China)
	Primary antibodies	*p*-P65	1:1000	Cell Signaling Technology (MA, USA)
	Primary antibodies	*p*-IκBα	1:2000	Affinity Biosciences (Cincinnati, OH, USA)
	Secondary antibodies	HRP-goat anti rabbit	1:50,000	Seracare (Milford, MA, USA)
	Secondary antibodies	HRP-goat anti mouse	1:50,000	Seracare (Milford, MA, USA)
